# Autophagy-dependent ferroptosis in kidney disease

**DOI:** 10.3389/fmed.2022.1071864

**Published:** 2023-01-23

**Authors:** Yuanting Yang, Jiayi Cheng, Qisheng Lin, Zhaohui Ni

**Affiliations:** ^1^Molecular Cell Lab for Kidney Disease, Department of Nephrology, Shanghai Peritoneal Dialysis Research Center, Uremia Diagnosis and Treatment Center, RenJi Hospital, Shanghai Jiao Tong University School of Medicine, Shanghai, China; ^2^Tianping Community Health Service Center, Shanghai, China

**Keywords:** ferroptosis, autophagy, kidney disease, cell death, acute kidney injury

## Abstract

Ferroptosis is a new type of cell death caused by the lack of glutathione peroxidase 4 (GPX4) and the imbalance of cellular redox. It is characterized by the accumulation of lipid peroxides on cell membranes. Multiple regulatory pathways of ferroptosis include the GPX4, glutamate-cystine antiporter (System Xc^–^), lipid metabolism, and iron metabolism pathways. Recent studies have reported that autophagy-dependent ferroptosis (ferroptosis meditated by ferritinophagy, lipophagy, and clockophagy) plays a significant role in the occurrence of several diseases, including diseases affecting the nerves, liver, lungs, and kidneys. This review provides an overview of research progress made on autophagy-dependent ferroptosis in kidney diseases.

## 1. Introduction

Cell death plays an essential role in normal development, maintenance of homeostasis, and prevention of hyperproliferative diseases, such as cancer. Recently, in addition to apoptosis, other types of non-apoptotic programmed cell death, such as necroptosis (1), pyroptosis (2), and ferroptosis, have been found (3). Ferroptosis, one of the newly discovered programmed cell death modes, is characterized by intracellular iron accumulation and lipid peroxidation. It is different from other cell death types, such as apoptosis, necrosis, and autophagic cell death, in morphology, biochemistry, and genetics (3). Under a transmission electron microscope, ferroptosis is manifested as decreased or disappearance of mitochondrial cristae and increased mitochondrial membrane density (4). Autophagy is a process of maintaining cellular homeostasis by lysosomal removal of excess proteins and destruction of organelles. Ferroptosis was previously considered to be independent of autophagy. However, in recent years, further studies on lipophagy, ferritinophagy, clockophagy, and chaperon-mediated autophagy have provided contrary evidence to suggest that autophagy may be required for the development of ferroptosis. Currently, autophagy has been found to promote the development of ferroptosis in several diseases, such as neurodegenerative diseases, liver fibrosis, and kidney diseases (5).

## 2. Ferroptosis

Dixon et al. (3) first discovered that erastin, a RAS selective lethal small molecule, can induce cell death, which is different from apoptosis, necrosis, and other types of programmed cell death. This lethal type of cell death could be prevented by iron chelators (DFO). Through screening and subsequent confirmation of LOC libraries, ferrostain-1(Fer-1) was found to be the most effective inhibitor of erastin-induced ferroptosis (3). Lipid peroxidation and iron overload are two central events in the biochemistry of ferroptosis.

### 2.1. Lipid peroxidation

Lipid peroxidation, an important characteristic of ferroptosis (6), is the target of polyunsaturated fatty acids (PUFAs). PUFAs include free PUFAs [such as arachidonic acid (AA) and adrenic acid (AdA)] (7) and PUFA-containing membrane phospholipids, such as phosphatidylethanolamines (PE) and phosphatidylcholine (PC). Dixon et al. (3) found that PE can be oxidized by acyl-CoA synthetase long chain family member 4 (ACSL4) and lysophosphatidylcholine acyltransferase 3 (LPCAT3) and further oxidized to lipid peroxides by lipoxygenase-15 (LOX-15). The accumulation of lipid peroxides on membranes causes rupture of cell membranes and release of cell contents to trigger ferroptosis. Therefore, lipid peroxides are considered to be the proximal executors of ferroptosis (8). Additionally, lipid peroxidation of PUFAs produces various oxidation products, such as malondialdehyde and 4-hydroxylnonenal (4-HNE), which are highly cytotoxic (9).

### 2.2. Iron overload

Iron contents are precisely regulated at both cellular and systemic levels to provide the iron needed for vital biological functions and prevent cytotoxicity resulting from iron overload (10). Although the theory that iron overload induces lipid peroxidation to promote ferroptosis through Fenton reaction remains controversial (6), intracellular iron overload as a necessary condition for ferroptosis has been proven. Before ferroptosis was discovered, Yang and Stockwell (11) and Yagoda et al. (12) found that the pretreatment of DFO to reduce intracellular iron could prevent erastin-induced cell death in HT1080 cells and BJ-TERT/LT/ST cells, respectively. Hou et al. (13) observed an increase in intracellular label iron pool (LIP) during the induction of ferroptosis in MEFs and HT-1080 cells. According to the study, the increase of iron content in ferroptosis is associated with upregulation of transferrin receptors (TfR) and downregulation of ferritin heavy chain 1 (FTH1) and ferritin light chains (FTL) (11).

## 3. Autophagy

Autophagy is the process of removing excess proteins and destroying organelles by lysosomal degradation. Three types of autophagy involving different modes of cargo delivery to lysosomes have been noted: macroautophagy, microautophagy, and chaperone-mediated autophagy.

### 3.1. Macroautophagy

During the process of macroautophagy, all organelles and some cytoplasmic contents are isolated in a double-membrane vesicle, which is known as an autophagosome. After the autophagosome matures, it fuses with lysosomes, forming an autolysosome. Subsequently, the organelles packaged by the autolysosome is degraded by lysosomal hydrolases (14). Autophagy plays a critical role in the development and progression of kidney disease and is often used to refer to macroautophagy, such as in mitophagy. According to Bhatia et al. (15), mitophagy is involved in renal cell survival and kidney function stabilization through the maintenance of mitochondrial homeostasis.

### 3.2. Microautophagy and chaperone-mediated autophagy

Unlike in macroautophagy, the autophagosome is not necessary for microautophagy and chaperone-mediated autophagy. In microautophagy, the invaginated lysosome can engulf the small bulk cytoplasm directly (16). The substrate proteins containing KFERQ-like pentapeptide sequence motifs are recognized by the chaperone protein, heat shock protein 70 (HSP70), and other complementary chaperone proteins in chaperone-mediated autophagy. Additionally, they are transported to lysosomes by forming a complex with lysosome-associated membrane protein (LAMP)-2A (17).

## 4. Autophagy-dependent ferroptosis

Ferroptosis was previously considered to be different from necrosis, apoptosis, and autophagy (3). However, due to evidence suggesting that autophagosomes are accumulated during the process of ferroptosis, this theory has been challenged. Moreover, several other evidences suggest that autophagic mechanisms, such as ferritinophagy, lipophagy, clockophagy, and chaperone-mediated autophagy, are required for the occurrence of ferroptosis (18, 19).

### 4.1. NCOA4-mediated ferritinophagy

Most of the iron contents are stored in ferritin, which is degraded by lysosomes to release free iron when needed in a process known as ferritinophagy. By proteomic analysis of purified autophagosomes, Mancias et al. (20) found that nuclear receptor coactivator 4 (NCOA4) is a ferritin transport carrier in ferritinophagy and the C-terminal element of NCOA4 can bind with arginine residue R23 on the surface of FTH1 to transport ferritin to autophagic lysosomes. The theory that NCOA4-mediated autophagic degradation of ferritin can increase cellular label iron pool (LIP) levels and promote the occurrence of ferroptosis has been confirmed in various disease models. Studies have shown that knockdown of NCOA4 or autophagy-related gene 5/7 (ATG5/ATG7) by shRNA in HT1080 cells can inhibit ferritinophagy in mouse embryonic fibroblasts, reduce the level of intracellular free irons, and inhibit the occurrence of ferroptosis. On the contrary, overexpression of NCOA4 promotes the occurrence of ferroptosis (13). Additionally, Kong et al. (21) and Zhang et al. (22) found that artesunate and RNA-binding protein, ELAVL1/HuR, could promote the occurrence of hepatic stellate cell ferroptosis by activating ferritinophagy, thereby aggravating liver fibrosis. Yoshida et al. (23) found that smoke exposure upregulated the level of ferritinophagy, promoted the occurrence of ferroptosis, and participated in the pathological process of chronic obstructive pulmonary disease. Therefore, NCOA4-mediated ferritinophagy plays a crucial role in promoting the pathogenesis of ferroptosis.

### 4.2. RAB7A-mediated lipophagy

The anabolic process of lipids occurs in the endoplasmic reticulum (ER) of hepatocytes. The accumulation of neutral lipids, such as triglycerides and cholesterol, on the ER leads to the formation of a spherical dynamic organelle known as lipid droplet (LD). The process by which lysosomes bind to LDs coated by autophagosomes and release free fatty acids (FFAs) is known as lipophagy (24). However, excessive lipolysis plays an important role in the occurrence of ferroptosis by increasing lipid toxicity and lipid peroxidation levels (25). RAB7A, a member of the RAS oncogene family, is a small molecule GTPase that recruits LDs and promotes LDs binding to lysosomes, which is essential in lipophagy (26). Studies have shown that shRNA knockdown of ATG5 or RAB7A to inhibit lipophagy and increase lipid storage can inhibit RSL3-induced ferroptosis in HepaG2 cells. On the contrary, shRNA knockdown of tumor protein D52 to inhibit lipid storage and upregulate lipophagy can promote the occurrence of ferroptosis (27).

### 4.3. Clockophagy (degradation of ARNTL)

The circadian clock system plays a key role in regulating various aspects of physiology, such as metabolism, organ function, hormone secretion, immunity, and cell cycle (28). Aryl hydrocarbon receptor Nuclear Translocator like protein 1 (ARNTL), a circadian clock transcription factor, is a core component of the mammalian clock system that regulates gene expression by binding to E-box sequences in promoters (29). In clockophagy, degradation of circadian clock protein, ARNTL, is mediated by Sequestosome1 (SQSTM1)/P62. It is a kind of selective autophagy in oxidative damage mediated by EGL9 family hypoxia-inducible factor 2/hypoxia-inducible factor prolyl Hydroxylase 1 (PHD1) (30). Yang et al. (30) found that inhibition of the degradation of ARNTL mediated by clockophagy could inhibit the occurrence of RSL3-induced ferroptosis by knocking down ATG5/ATG7 or SQSTM1 and the depletion of ARNTL-aggravated lipid peroxidation. ARNTL can inhibit the degradation of hypoxia-inducible factor 1-α (HIF1-α) by EGLN2, thereby regulating the redox balance and inhibiting the occurrence of ferroptosis (30).

### 4.4. Other types of autophagy

**FIGURE 1:**
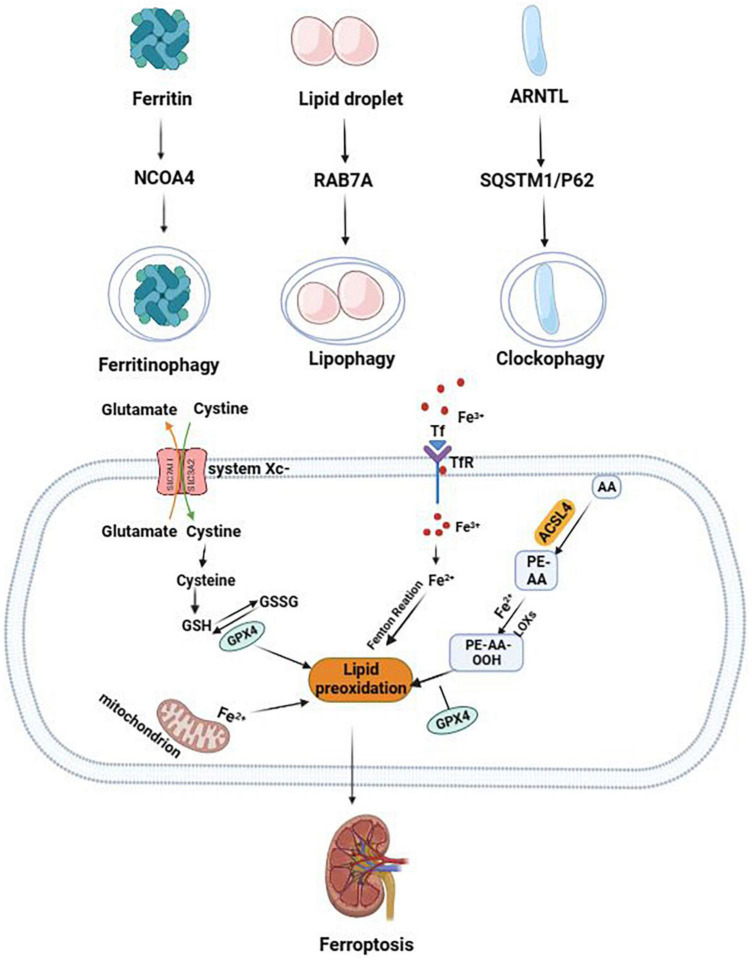
The mechanism of autophagy-dependent ferroptosis. The two central events in the biochemistry of ferroptosis are lipid peroxidation and iron overload. NCOA4-mediated ferritinophagy promotes the occurrence of ferroptosis by upregulating free iron level. RAB7A-mediated lipophagy promotes the occurrence of ferroptosis by increasing lipid toxicity and lipid peroxidation levels. Clockophagy (degradation of ARNTL) promotes the occurrence of ferroptosis by regulating the redox balance.

In addition to lipophagy, ferritinophagy, and clockophagy, other autophagy mechanisms or autophagy-related molecules are involved in the occurrence of ferroptosis. BECN1 can inhibit the activity of XC-system by binding to Solute Carrier family 7 member 11 (SLC7A11) and promote the occurrence of erastin-induced ferroptosis (31). Wu et al. (32) found that increased iron levels in ferroptosis caused increased levels of lysosome-associated membrane protein 2A (LAMP-2A) and chaperone-mediated autophagy (CMA), leading to degradation of CMA substrate antioxidant protein, GPX4, promoting the occurrence of ferroptosis in HT-22 cells (Figure 1).

## 5. Regulatory pathways of ferroptosis

### 5.1. Glutathione peroxidase 4 pathway

Dixon et al. (3) found that RSL3 treatment did not prevent the input of radio-labeled cystine and the expression of glutathione (GSH) was not affected, although RSL3 was widely considered as the inducer of ferroptosis. By mass spectrometry proteomics analysis, the target of RSL3 was determined to be the glutathione peroxidase 4 (GPX4) pathway (33). GPX4 can use GSH as a reducing cofactor to reduce lipid peroxides to alcohols, regulating redox balance and protecting cell membranes from peroxides damage. Studies have shown that RSL3 treatment, by inhibiting GPX4 activity, promotes the increase of the label iron pool and accumulation of lipid ROS, inducing the occurrence of ferroptosis (34).

### 5.2. System Xc^–^ pathway

The glutamate-cysteine anti-transport system (System Xc^–^) consists of 12 transmembrane transporters (Solute Carrier family 7 member 11, SLC7A11) and single-channel transmembrane regulatory proteins (Solute Carrier family 3 member 2, SLC3A2) (35), which can be transferred at a ratio of 1:1 out of glutamic acid and into cystine in an ATP-dependent manner (3). Studies have found that erastin can directly bind to SLC7A11 to inhibit the activity of XC-system, prevent the import of cystine, and inhibit the synthesis of intracellular GSH (36). Additionally, recent studies have shown that the treatment of sorafenib in hepatocellular carcinoma can promote the occurrence of ferroptosis in cancer cells by SLC7A11-dependent blockage of GSH synthesis (37).

### 5.3. Lipid metabolism pathway

Lipid molecules in cells have various functions, such as signaling, energy storage, and basic functions as bio-membrane building molecules. Enzymes involved in lipid metabolism, such as ACSL4 and LPCAT3, are critical in the regulation of ferroptosis. Doll et al. (38) used CRISPR-based whole genome gene screening and microarray analysis of ferroptosis-resistant cell lines to reveal ACSL4 as an important inducer of ferroptosis. Additionally, the study found that Gpx4/Acsl4 double knockout cells had unexpected resistant properties to ferroptosis. Dixon et al. (8) used human haploid cytogenetics to reveal that the lipid metabolism genes, ACSL4 and LPCAT3, can promote the occurrence of lipid peroxidation.

### 5.4. Iron metabolism pathway

Iron metabolism processes, such as uptake, storage, utilization, and excretion, regulate the occurrence of ferroptosis. Dixon et al. (3) found that silencing iron response element binding protein 2 (IREB2) with shRNA could inhibit iron metabolism-related protein genes, such as transferrin receptor-related gene (TFRC), FTH1, and FTL, inhibiting the occurrence of ferroptosis. At low levels of iron, arginine residue, R23, on the surface of FTH1 can combine with NCOA4 on lysosomes, releasing free iron to replenish iron levels (39). Yoshida et al. (23) found that knocking down NCOA4 gene, inhibiting ferritinophagy, and increasing iron storage could inhibit ferroptosis induced by smoke exposure in chronic obstructive pulmonary disease. Therefore, the expression level of genes or proteins involved in iron metabolism affects the sensitivity of cells to ferroptosis.

## 6. The detection of autophagy-dependent ferroptosis

First, the detection of ferroptosis is unnecessary for autophagy-dependent ferroptosis. For example, BODIPY™ 581/591 C11 staining to evaluate lipid peroxidation and ferroptosis key molecules, including GPX4, GSH, and ACSL4, to evaluate the level of ferroptosis in cells. Additionally, the expression level of two subunits of ferritin, FTH1 and FTL, and NCOA4, the transportation carriers of ferritin, should be assessed, and the level of intracellular free iron is essential for detecting ferritinophagy. Co-localization of immunofluorescent FTH1 and NCOA4 with autophagosomes is most important. For lipophagy, the lipid droplets coated by a double membrane structure can be observed by transmission electron microscopy. Additionally, lipophagy can be monitored by total lipid levels using BODIPY 493/503 or cell staining with Oil Red O. The co-localization of ARNTL/BMAL1 and autophagosome can be monitored under a confocal microscope for clockophagy (40).

## 7. Autophagy-dependent ferroptosis and kidney disease

### 7.1. Chronic kidney disease

The incidence of chronic kidney disease (CKD) is 8–16% among the global population (41). CKD is a clinical syndrome with glomerular filtration rate < 60 mL/min/1.73 m^2^, proteinuria > 30 mg/d, or renal injury markers lasting > 3 months. It is caused by various conditions; the most common causes are diabetes and hypertension. Zhou et al. (42) found that typical features of ferroptosis, such as downregulation of GPX4 expression and increase in 4-HNE level, were observed in CKD mouse models. In these models, Fer-1 treatment alleviated renal injury and fibrosis. Additionally, Jin and Chen (43) found that in diabetic nephropathy (DN), umbelliferon significantly improves renal injury and ROS generation by activating the Nrf2/HO-1 pathway to inhibit ferroptosis, thus playing a protective role in DN. According to Wang et al. (44), the CKD rat models showed typical features of ferroptosis, such as increased iron content, oxidative stress, and lipid peroxidation. Further studies found that the expression of NCOA4 was upregulated and the expression of FTH1 and FTL was downregulated in the residual kidney tissue, which was reversed after DFO treatment. This finding indicated that the occurrence of ferroptosis in the CKD group was related to iron overload caused by ferritinophagy.

According to these studies, the progression of CKD cannot be separated from ferroptosis. Downregulation of intracellular free iron by inhibiting ferritinophagy and ferroptosis may be an effective therapeutic target for CKD.

### 7.2. Acute kidney injury

Acute kidney injury (AKI) is a clinical syndrome caused by sepsis, hypovolemia, contrast media, or other nephrotoxic drugs. It is characterized by a rapid decline of renal function in a short period of time, resulting in a dysregulation of the body’s internal environment (azotemia, water, electrolyte, and acid-base balance disorders, etc.) (45). The occurrence and development of AKI are often accompanied by the death of renal tubular cells, which is involved in various cell death modes, including necrosis, apoptosis (46), ferroptosis (47), and autophagic cell death (48). From our previous study findings, in contrast-induced acute kidney injury (CI-AKI), PINK1/PARK2-mediated mitophagy downregulated ROS-mediated DNA oxidative damage and mitochondrial ROS generation in renal tubular epithelial cells to maintain the redox balance of tubular epithelial cells (48). Based on the above studies, we hypothesized that autophagy regulated redox balance and affected the development of ferroptosis. Studies on folic acid-induced acute kidney injury (FA-AKI) revealed that lipid peroxidation, GPX4 depletion, and the application of Fer-1 can protect renal function, and reduce tissue damage and renal tubular cell death caused by folic acid (49). Another study showed that lipid peroxidation and ROS generation were significantly increased in cisplatin-induced acute kidney injury (cis-AKI), and the expression level of GPX4 was downregulated in Vitamin D receptor knockout mice, promoting the occurrence of ferroptosis in renal tubular epithelial cells and aggravating renal tissue injury caused by cisplatin (50). Additionally, autophagy-dependent ferroptosis is involved in the pathological process of AKI. Chen et al. (51) found that lipid preoxidation and ferroptosis were decreased, but expression of GPX4 was increased in ischemia-reperfusion acute kidney injury (IRI) mice treated with legumain knockout in a study on IRI in 2021. Further studies showed that legumain could promote the degradation of GPX4 by lysosomal activities and promote the occurrence of ferroptosis by combining with HSP70 and LAMP-2A. Cold inducible RNA binding protein (CIRBP), a regulator of inflammatory responses, is usually low and increased under stress conditions, hypoxia, and ischemia-reperfusion. According to Sui et al. (52), CRIBP promotes renal injury in IRI by upregulating ferroptosis, and silencing CRIBP can inhibit the occurrence of ferroptosis in HK-2 cells. Further studies revealed that CRIBP activated ferritinophagy by interacting with the RNA-binding protein, ELAVL1, and promoted the occurrence of ferroptosis.

### 7.3. Urinary tract infections (UTIs)

UTIs are one of the most common bacterial infections in the community and healthcare system (53). More than 25% of UTI cases recur, resulting in the persistence of drug-resistant strains (54). The study has shown that ferritinophagy is activated in uropathogenic *E. coli* (UPEC)-infected bladder epithelial cells (BECs) treated with ferric ammonium citrate (FAC), causing the persistence of UPEC in BECs and increasing the risk of recurrence and reinfection. Additionally, FAC treatment promoted the death of host BECs while activating ferritinophagy. It is neither apoptosis nor pyroptosis, but a kind of cell death caused by iron overload. However, the authors did not examine the phenotype of ferroptosis (55).

In conclusion, the persistence of UPEC in BECs is involved in ferritinophagy, which promotes the death of BECs and aggravates UTIs. The inhibition of ferritinophagy and downregulation of iron content in BECs may serve as a potential therapeutic target for the control of UPEC growth and provide a theoretical basis for the prevention of UTI recurrence and reinfection.

### 7.4. Clear cell renal cell carcinoma (ccRCC)

ccRCC is the most common and aggressive subtype of renal cell carcinoma, accounting for about 70–80% of renal cell carcinoma cases (56). Zou et al. (57) found the mechanism of targeted inhibition of GPX4 expression in the treatment of ccRCC by CRISPR screening and lipidomic analysis. HIF1/2-α can selectively recruit lipid droplets by activating hypoxia-inducible lipid droplet-associated protein (HLDAP), promote lipid droplet decomposition, upregulate the content of PUFAs in cells, and increase the sensitivity of cancer cells to ferroptosis. However, the authors did not verify autophagy-related phenotypes of lipid droplets. Additionally, Mou et al. (58) found in a 2021 study that the expression level of NCOA4 mRNA in tumor cells of patients with ccRCC was significantly lower than that of normal tissues in TGGA database and GEO database (*p* = 7.337e-2, *p* = 4.696e-050.018), which is associated with overall survival (OS) and immune cell infiltration in patients with ccRCC. Further studies found that increased FTH1 levels were associated with poorer prognosis in patients with ccRCC. Since FTH1 and NCOA4 are important proteins related to ferritinophagy, tumor cells of patients with ccRCC may block the autophagy degradation pathway of ferritin in lysosomes through the low expression of NCOA4, downregulate the level of label iron pool in tumor cells, and inhibit the occurrence of ferroptosis to achieve immune evasion of tumor cells.

According to the above studies, inhibiting GPX4 to induce ferroptosis in renal cancer cells may be a breakthrough for future applications in cancer therapeutics. Concurrently, inhibiting the degradation of NCOA4, enhancing the level of ferritinophagy, and promoting the occurrence of ferroptosis in renal cancer cells can provide a theoretical basis for the treatment of renal cancer.

## 8. Discussion

### 8.1. Conclusion

Based on the current studies, the role of autophagy-dependent ferroptosis cannot be ignored in kidney diseases and renal carcinoma. However, the role of autophagy-dependent ferroptosis in other types of kidney diseases has not been reported. Therefore, further studies are needed to provide a theoretical basis and guidance for targeted therapy of kidney diseases and renal carcinoma.

### 8.2. Limitations

Autophagy-dependent ferroptosis is an innovative theory, which has only been validated in experimental animals and cellular experiments, and relevant clinical studies are lacking. This review is a summary of current international studies or data on autophagy-dependent ferroptosis to guide future scientific research. Further exploratory studies are needed to assist in clinical decision-making and application.

## Author contributions

YY wrote the manuscript. JC helped interpret data and figure. QL and ZN edited the manuscript. All authors contributed to the article and approved the submitted version.
